# Estrogen Deficiency Impairs Osseointegration in Hypertensive Rats Even Treated with Alendronate Coated on the Implant Surface

**DOI:** 10.3390/jfb14090471

**Published:** 2023-09-13

**Authors:** Gabriel Mulinari-Santos, Jaqueline Silva dos Santos, Igor Lebedenco Kitagawa, Fábio Roberto de Souza Batista, Paulo Roberto Botacin, Cristina Antoniali, Paulo Noronha Lisboa-Filho, Roberta Okamoto

**Affiliations:** 1 Department of Diagnostic and Surgery, School of Dentistry, São Paulo State University (UNESP), Araçatuba, SP, Brazil; 2 Department of Basic Science, School of Dentistry, São Paulo State University (UNESP), Araçatuba, SP, Brazil; 3 Federal Institute of Education, Science and Technology of São Paulo (IFSP), Birigui, SP, Brazil; 4 Department of Physics, School of Science, São Paulo State University (UNESP), Bauru, SP, Brazil

**Keywords:** bone, losartan, osseointegration, alendronate, spontaneously hypertensive rats

## Abstract

Hypertension and estrogen deficiency can affect bone metabolism and therefore increase the risk of osseointegration. Antihypertensive drugs such as losartan not only control blood pressure but also enhance bone healing. In addition, alendronate sodium is widely used to treat postmenopausal osteoporosis. Hence, we evaluated the effect of systemic antihypertensive and local alendronate coted on implants on osseointegration under hypertensive and estrogen-deficiency conditions. A total of 64 spontaneously hypertensive rats (SHRs) treated with losartan were randomly divided according to the estrogen-deficiency induction by ovariectomy (OVX) or not (SHAM), and whether the implant surface was coated with sodium alendronate (ALE) or not, resulting in four groups: SHR SHAM, SHR SHAM ALE, SHR OVX, and SHR OVX ALE. The removal torque, microcomputed tomography, and epifluorescence microscopy were the adopted analyses. The hypertensive and estrogen-deficiency animals presented a lower removal torque even when treated with alendronate on implant surface. The microcomputed tomography revealed a higher bone volume and bone-to-implant contact in the SHRs than the SHR OVX rats. Epifluorescence showed a decreased mineral apposition ratio in the SHR OVX ALE group. The data presented indicate that estrogen deficiency impairs osseointegration in hypertensive rats; in addition, alendronate coated on the implant surface does not fully reverse this impaired condition caused by estrogen deficiency.

## 1. Introduction

Hypertension and osteoporosis are the major causes of mortality and morbidity in public health, especially due to the increased risks for cardiovascular diseases and bone fractures [1]. These systemic alterations are more common in postmenopausal women; moreover, hypertensive females develop osteoporosis more frequently [2]. Osteoporotic patients are also more associated with hypertension, and there is a possible relation between hypertension and osteoporosis in diets with low calcium intake [2], mainly due to estrogen deficiency [3]. Hypertension and estrogen deficiency are responsible for promoting bone metabolism changes [4,5,6,7]. Thus, both of these associated conditions may be at high risk for impaired osseointegration. Despite the elevated frequency of postmenopausal hypertensive individuals, peri-implant bone healing under hypertension and estrogen deficiency remains uncertain. At least, therapeutic strategies must be adopted to improve this compromised bone metabolism.

Antihypertensive drugs act to suppress the renin–angiotensin system, prevent vasoconstriction, and control blood pressure [8]. Additionally, recent studies indicate antihypertensive drugs are capable of acting on the bone metabolism [9,10,11]. Components of the renin–angiotensin system play a role in bone [9,12]. Consequently, angiotensin II (Ang II) increases the bone resorption of osteoclasts by increasing the receptor activator of nuclear factor-κB ligand (RANKL) [9,10,11]. Furthermore, Ang II diminishes osteoblasts, reducing the expression of runt-related transcription factor 2 (RUNX2) and osteocalcin [13]. Conversely, losartan is an angiotensin II receptor type 1 antagonist widely used to control hypertension [14], which also impacts the bones [9,10,11]. Anticatabolic effects, such as improving the mechanical properties of the alveolar bone [15] and decreasing the risks of fractures from osteoporosis, were found [16,17]. In preclinical studies, losartan has improved bone mineralization and fracture healing [13,18], corroborating with clinical studies where antihypertensive drugs were stated as responsible for increasing the survival of osseointegrated implants [19,20]. Thereby, losartan may provide benefits for osseointegration in compromised bone conditions by hypertension and estrogen deficiency.

Alendronate sodium is a drug extensively used for osteoporosis; it consists of second-generation bisphosphonate, classified as an antiresorptive agent [21,22]. This drug can decrease bone fractures and improve alveolar bone healing [23]. Recent research has shown increased osseointegration parameters in osteoporosis models treated with alendronate [24]. However, the clinical use of alendronate is still imprecise, since there are side effects, such as nausea, vomiting, gastrointestinal pain [25], and osteonecrosis of the jaws, after long-term systemic use [26]. For reducing side effects, alendronate administrated locally has being one of the most promising therapies [23,24,27]. When it was applied on the implant surface, alendronate improved the values of bone volume and bone–implant contact [24], as well as in experimental osteoporosis [28]. Therefore, the local use of alendronate coated on the implant surface can be a beneficial treatment strategy for implant placement in hypertensive females with estrogen deficiency. Given the high incidence of hypertension and osteoporosis especially in females [4], the model of estrogen deficiency by ovariectomy in spontaneously hypertensive rats was proposed to simulate this challenging clinical condition for rehabilitation with osseointegrated implants.

## 2. Materials and Methods

### 2.1. Study Design and Ethics

This study was sent to the Animal Research Ethics Committee and obtained a favorable opinion under number 00332-2018. All research procedures have been established according to the ARRIVE guidelines [29]. A total of 64 female spontaneously hypertensive rats (SHRs), 6 months old, weighing around 300 g, were used. The animals were preserved in cages in a constant temperature environment (22 °C ± 2 °C; light control cycle of 12 light hours and 12 h dark) and provided a balanced diet (1.4% Ca and 0.8% Pe, Nuvilab, Curitiba, PR, Brazil). The rats were separated into four groups according to the estrogen-deficiency induction by ovariectomy surgery (OVX) or not (SHAM), and whether the implant surface was treated with sodium alendronate (ALE) or not, resulting in: SHR SHAM, SHR SHAM ALE, SHR OVX, and SHR OVX ALE. The randomization list was created with a computer-generated list in Stata 9.0 (StataCorp, College Station, TX, USA).

### 2.2. Strus Cycle and Ovariectomy Surgery

Before the ovariectomy surgery, it was checked whether the hypertensive rats were cycling normally. Daily, they were placed in individual cages and 1–2 drops of saline were introduced into the vagina, then extracted and positioned on a histology slide for microscopic reading to recognize the phases of the estrous cycle, using the Long and Evans technique [30]. Rats were used after obtaining 2 to 3 regular estrous cycles. Surgical removal of both ovaries was performed under anesthesia with 100 mg/kg of ketamine (Vetaset—Fort Dodge Saúde Animal Ltda., Campinas, São Paulo, Brazil) intramuscularly and 5 mg/kg of xylazine (Dopaser—Laboratório Calier do Brasil Ltda.—Osasco, São Paulo, Brazil) intraperitoneally.

### 2.3. Losartan Treatment and Systolic Blood Pressure

Losartan (Biosintetica, São Paulo, Brazil) was administered orally in all groups at concentrations of 30 mg/kg/day until euthanasia. The losartan treatment started 30 days after the ovariectomy and 7 days before the implant surgery, and continued after implant placement, extending until the euthanasia. Systolic blood pressure was checked immediately prior to the implant placement to ensure that the blood pressure was controlled below 150 mmHg. This verification was used to simulate a real control condition, since uncontrolled hypertension preoperatively contraindicates implant surgery. Here, we used the tail-cuff indirect plethysmography method with a Physiograph^®^ MK-III-S (Narco Bio-Sistemas, Houston, TX, USA) according to a prior study [31].

### 2.4. Treatment of Implants with Sodium Alendronate

The implants were coated using the layer-by-layer technique, in which layers of polyelectrolyte were added via electrostatic interaction. For a monolayer of alendronate sodium coating on the implant’s surface, implants were first immersed in a layer of sodium polystyrene sulfate. Then, the implants were immersed in a layer of titanium dioxide; finally, the implants were coated with a layer of sodium alendronate at a concentration of 1 g using electrostatic attraction. The suspension ingredients were blended in accurate quantities in water-cooled glass vials with a sonicator probe (model XLS-2015, Misonix, Inc., New York, NY, USA). Each step of the implant’s coating layer followed a previously described method [32].

### 2.5. Implant Placement

The implant placement followed an earlier successful bicortical rat-tibia-implant model [33,34]. For the surgery to place the implants in the tibias, the animals underwent preoperative fasting for 8 h to be anesthetized with a combination of 100 mg/kg of ketamine (Vetaset—Fort Dodge Saúde Animal Ltda., Campinas, São Paulo, Brazil) intramuscularly and 5 mg/kg of xylazine (Dopaser—Laboratório Calier do Brasil Ltda.–Osasco, São Paulo, Brazil). Mepivacaine hydrochloride (0.3 mL/kg 2% Scandicaine, adrenaline 1: 100,000; Septodont, Saint-Maur-des-Fosse’s, France) was administered for anesthesia and hemostasis. After anesthesia, the surgical site in the medial tibial portion was shaved and subjected to topical disinfection with degerming iodine (10% PVPI, Riodeine degermante; Rioquímica, São José do Rio Preto, SP, Brazil). A 1.5 cm incision was performed, followed by divulsion to expose the tibial metaphysis. A grade-four titanium implant treated with aluminum oxide and nitric acid (HNO3, Mendes, Itu, São Paulo, Brazil) was installed in the SHR SHAM and SHR OVX groups, with dimensions of 2 mm in diameter and 4 mm in length. The SHR SHAM ALE and SHR OVX ALE groups received the same implants with alendronate incorporated into their surfaces. With the 1.6 mm diameter reamer coupled to the electric motor (BLM 600, Driller, São Paulo, SP, Brazil) at a speed of 1000 RPM under isotonic irrigation of 0.9% sodium chloride (Fisiológico, Laboratórios Biosintética Ltda., Ribeirão Preto, SP, Brazil), the surgical defect was created for subsequent manual installation of the implant with a hexagonal digital driver. The wound was sutured in layers, with the deep layer using absorbable thread (Vycril 4.0, Ethicon, Johnson, São José dos Campos, Brazil) and the outer layer with monofilament (Nylon 5.0, Ethicon, Johnson, São José dos Campos, Brazil). In the immediate postoperative, the animals received an injection of intramuscular pentabiotic (0.1 mg/kg, Fort Dodge Saúde Animal Ltda., Campinas, São Paulo, Brazil) and sodium dipyrone (1 mg/kg/1 day ^−1^, Ariston Indústrias Químicas e Farmacêuticas Ltda., São Paulo, Brazil).

### 2.6. Fluorochrome Application

Twenty-one days after starting losartan treatment, fluorochrome calcein was administered intramuscularly (20 mg/kg, Sigma Chemical Company, St. Louis, MI, USA). In addition, alizarin red fluorochrome was used intramuscularly after 28 days (20 mg/kg, Sigma Chemical Company, St. Louis, MI, USA). The earliest infused calcein fluorochrome implied calcium deposition in the old bone, and later, the alizarin fluorochrome in the newly-formed bone, as per a previous study [31,33].

### 2.7. Euthanasia

All animals were euthanized 60 days after implant installation using a lethal dosage of anesthetic (60 mg/kg; Tiopental Cristália, Ltda., Itapira, SP, Brazil).

### 2.8. Analysis

#### 2.8.1. Biomechanical Test

Removal torque was measured using an implant hexagon and a digital torque (torque meter, Conexão, São Paulo, Brazil). Then, the removal torque of eight tibias per subgroup was registered with an anticlockwise movement utilized by increasing the force for the removal torque until the implant turned in the bone at the highest torque point in Newton centimeters [34].

#### 2.8.2. Computed Microtomography (MicroCT)

The tibias of eight rats per subgroup were removed after euthanasia, fixed in a 10% formalin solution (Analytical Reagents^®^, Dinâmica Odonto-Hospitalar Ltda., Catanduva, SP, Brazil) for 48 h, bathed in running water for 24 h, and stored in 70% alcohol to be transported until scanned. For scanning, SkyScan microtomography (SkyScan 1272 Bruker MicroCT, Aatselaar, Belgium, 2003) was used with 9 µm thick slices (50 Kv and 500 μ), copper and aluminum filters, and a rotation pitch of 0.3 mm. The sequence of images obtained was three-dimensionally reconstructed using the NRecon software (SkyScan, 2011; Version 1.6.6.0). In the Data Viewer software (SkyScan, Version 1.4.4 64-bit), the positioning of all samples in the transverse, longitudinal, and sagittal planes was standardized. Then, in the CTAnalyzer software (2003-11SkyScan, 2012 Bruker MicroCT Version 1.12.4.0), the analysis region around the implant was defined in the transverse plane. They were marked at 50 slices in a proximal direction and 50 slices in a distal direction in the bone in which the implant was installed, thus totaling a volume of 100 slices (874.3 µm). The CTAnalyzer software calculated the morphometric value based on the grayscale (threshold). The threshold used in the analysis was 25–90 shades of gray to delimit the bone formed around the implants. From the conversion, the software carried out the morphometric calculation applied to the implant in a transverse direction and provided the parameters of the bone volume percentage (BV/TV), the amount of bone in contact between the bone and implant (BIC), the trabecular thickness (Tb.Th), the number of trabeculae (Tb.N), the trabecular separation (Tb.Sp), and the total porosity (PO TOT). The adopted parameters followed the guidelines provided by the American Society of Bone and Mineral Research [35] and analyses carried out in previous studies [34]. The microtomographic reconstruction of each of the samples was performed using the CTvox software (SkyScan, Version 2.7).

#### 2.8.3. Epifluorescence Microscopy

After fixation in formaldehyde 10% for 48 h, the pieces were washed for 24 h in continuously running water and dehydrated in a gradually increasing order of alcohols. The specimens were included in a solution at a 1:1 ratio of acetone and methylmethacrylate (Classical, Dental Articles Classical, São Paulo, SP, Brazil), followed by methylmethacrylate baths. In the final bath was added benzoyl peroxide (1%, Riedel-de Haen AG, Seelze-Hannover, Germany). Samples were kept in glass tubing at 37 °C for five days. After polymerization, resin blocks holding the samples were detached from the glass cylinders. Then, the blocks were cut down to the implant along the tibia longitudinal plane with a drill mounted on an electric motor (Strong 210, São Paulo, SP, Brazil). Later, the samples were ground on politriz (Ecomet 250 pro automate 250, Buchler, Lake Bluff, IL, USA) with sandpaper (120, 300, 400, 600, 800, and 1200 granules, Carbimet, Buchler, Lake Bluff, IL, USA) to the thickness of 80 μm, checked by a digital caliper (Mitutoyo, Pompeia, São Paulo, Brazil). The pieces were mounted on slides submerged in mineral oil (Petrolato liquid, Maantecor, Taquara, Rio de Janeiro, Brazil).

Later, once the slides were ready, it was possible to capture images of the peri-implant bone using the confocal laser microscope Leica CTR 4000 CS SPE (Leica Microsystems, Heidelberg, Germany), using a 10X objective (Original magnification of 100×). Figures were recreated in a microscope software (Leica CTR 4000 CS SPE, Leica Microsystems, Heidelberg, Germany). The blue filter was used to visualize the calcein fluorochrome, shown in green color. The alizarin fluorochrome was revealed in red by the green filter. The scanned images were analyzed with the Image J software version 1.53 (Image Analyzer Software, Ontario, ON, Canada). The tool “color selection” was used on each image for standardizing the hue, saturation, and brightness. The peri-implant bone was detected in the same location on a unique slide to measure the mineral apposition rate. The daily value of mineralization was calculated by the distance between the calcein and alizarin, divided by the 28 days among their applications, resulting in the mineral apposition rate, as previously reported [31].

### 2.9. Statistics

The statistics were performed in the GraphPad Prism 9 software (GraphPad Software; La Jolla, CA; USA). Having obtained all data, first, the homoscedasticity test (Shapiro–Wilk) was performed, considering *p* < 0.05 to confirm the distribution of the data in the normality curve. Thus, the two-way ANOVA test was applied for two independent variables, such as the systemic condition (SHAM vs. OVX) and the treatment surface with ALE or not, and one dependent variable, such as the removal torque in Newton per centimeter, as well as each microtomography parameter (BV/TV; BIC; Tb.Th; Tb.N; Tb.Sp; PO TOT), mineral apposition rate value in micrometers, and the systolic blood pressure. In case of a significant relational effect, Sidak’s multiple comparisons test was directed to test all multiple-treatment groups.

## 3. Results

### 3.1. Systolic Blood Pressure

After 7 days of treatment with losartan, all animals obtained the systolic blood pressure below 150 mmHg preoperatively. This key feature of the target was determined since untreated hypertensive rats exhibit blood pressure levels above this threshold [31,36,37]. This adopted value reflects the effectiveness of the reduction in blood pressure by losartan with measurements based on prior studies [31,37]. Antihypertensive drugs have previously demonstrated to be capable of reducing the blood pressure of hypertensive animals from 171.4 ± 0.6 to 129.3 ± 0.9 mmHg when treated with losartan [31] and from 159.91 ± 7.09 to 114.07 ± 8.07 mmHg when treated with captopril [37]. Therefore, after confirmation that losartan effectively managed the systolic blood in the preoperative, all animals followed to the implant-placement surgery.

### 3.2. Removal Torque Values

**Figure 1:**
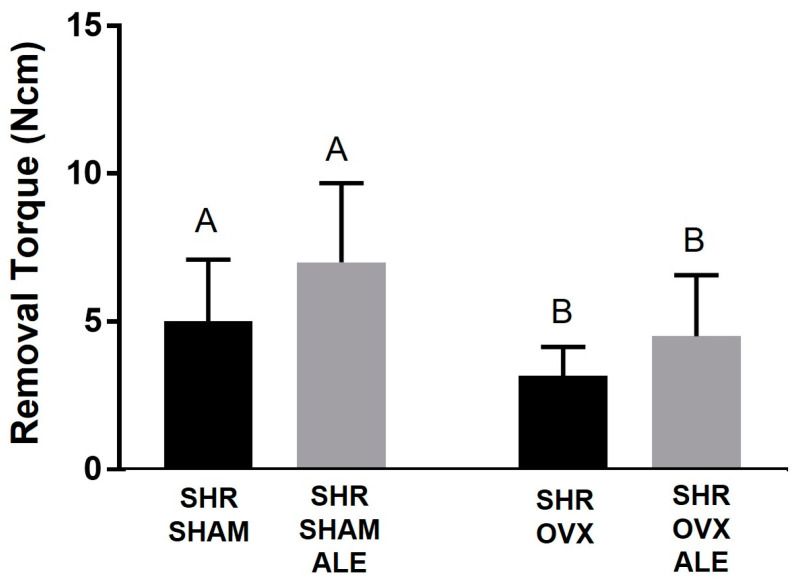
Biomechanical test of removal torque. Sixty days following implant installation, the removal torque for each experimental group was: SHR SHAM, SHR SHAM ALE, SHR OVX, and SHR OVX ALE. The removal torque was augmented until the implant rotated inside the bone and the highest torque in Newton centimeter (Ncm) was registered. The different uppercase letters indicate significant statistical differences in the systemic condition of the ovariectomy, comparing both SHAM groups to OVX groups (*p* < 0.05).

Sixty days after implant installation, the torque was measured for all experimental groups (Figure 1). Before the removal torque test, it was observed clinically that all animals exhibited stable consolidation of the implants in their tibias. The successful installation of the implants was confirmed by the removal test and subsequent microtomography analysis, which showed that all implants demonstrated biomechanical resistance and peri-implant osteogenesis.

The removal torque increased until the implant turned within the bone, and the peak strength value was recorded. In evaluating the values, the SHR SHAM ALE group obtained the highest average of 7.2 Ncm. The SHR SHAM group achieved an average of 5 Ncm, and the SHR OVX ALE group was 4.7 Ncm. The SHR OVX group obtained the lowest value with 3.33 Ncm. Having these data, first, the homoscedasticity test (Shapiro–Wilk) was performed considering a *p* < 0.05. Thus, the two-way ANOVA test was applied for two independent variables, the systemic condition and treatment surface, and one dependent variable, the removal torque. A statistical difference was observed between the systemic condition in the SHAM vs. OVX groups (*p* = 0.017).

Thereby, it was first determined whether hypertension and estrogen deficiency negatively affected the resistance against removal by the torque wrench. In support of this hypothesis, the removal torque was significantly lower in both the SHR OVX and SHR OVX ALE groups compared to both the SHR SHAM and SHR SHAM ALE groups (3.33 ± 0.1 Ncm and 4.7 ± 0.3 Ncm vs. 5.0 ± 0.2 Ncm and 7.2 ± 0.3 Ncm; *p* = 0.017). In the SHR OVX ALE group, the local alendronate had a tendency toward an elevation in the removal torque; however, it did not achieve significance (*p* = 0.55). The biomechanical test exposed the negative impacts of estrogen deficiency caused by ovariectomy on implant biomechanical stability in hypertensive rats (Figure 1).

### 3.3. Microcomputed Tomography Results

The effects of untreated hypertension on the analyzed parameters of microtomography are already reported in the literature [34]. Untreated hypertension has shown a peri-implant bone thickness of 0.096 ± 0.003 mm and a BV/TV of about 45% in hypertensive rats [34]. These data were used as a starting point for the present experimental design.

**Figure 2:**
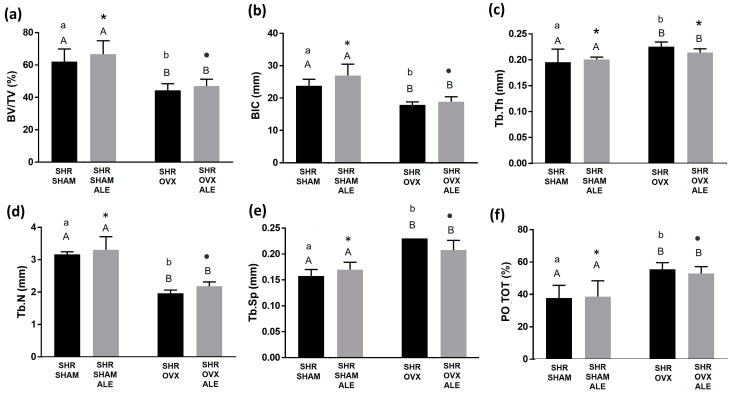
Microcomputerized tomography of peri-implant bone. Morphologic parameters were calculated and reported as: (**a**) Bone volume per tissue volume (BV/TV); (**b**) Bone-to-implant contact; (**c**) Trabecular thickness (Tb.Th); (**d**) Trabecular number (Tb.N.); (**e**) Trabecular separation (Tb.Sp); (**f**) Total porosity. The different uppercase letters indicate a significant statistical difference (*p* < 0.05) between systemic conditions. The different lowercase letters denote significant statistical differences (*p* < 0.05) between the SHR SHAM and SHR OVX groups with no alendronate surface. The alteration of symbols “*” and dot represents statistical differences (*p* < 0.05) between the SHR SHAM ALE and SHR OVX ALE groups.

Consistent with the biomechanical outcomes, the systemic condition of estrogen deficiency by ovariectomy significantly decreased the mean BV/TV in the hypertensive and ovariectomized rats of both the SHR OVX and SHR OVX ALE groups compared to the SHR SHAM and SHR SHAM ALE groups (45.6% ± 0.6% and 47.9% ± 0.4% vs. 61.5% ± 0.6% and 64.8% ± 0.6%; *p* < 0.0001; two-way ANOVA). Also, a statistical difference was noted between the alendronate groups of the SHR SHAM ALE and SHR OVX ALE groups (*p* = 0018; Sidak’s test). Additionally, the SHR SHAM and SHR OVX groups with conventional surfaces were statically different (*p* = 0.0039; Sidak’s test), reinforcing the systemic condition, and not the implant’s alendronate surface, as the major cause of the difference when comparing the groups (Figure 2a).

Accordingly, the BIC demonstrated the highest value for the SHR SHAM ALE group, followed by the SHR SHAM group (25 mm ± 0.6 and 22 mm ± 0.4), and both were statistically different with both OVX groups (*p* < 0.0001; two-way ANOVA). A statistical difference between the alendronate groups of SHR SHAM ALE and SHR OVX ALE was obtained (*p* = 0.004; Sidak’s test). Also, the SHR SHAM and SHR OVX groups with conventional surfaces were statically different (*p* = 0005; Sidak’s test; Figure 2b).

Possibly, the catabolic effect of estrogen deficiency on BV/TV obtained a trend to recompense by increasing the thickness of the bone trabeculae in the SHR OVX and SHR OVX ALE animals compared to the SHR SHAM and SHR SHAM ALE controls (0.22 ± 0.149 mm and 0.21 ± 0.198 mm vs. 0.019 ± 0.003 mm and 0.019 ± 0.003 mm; *, *p* = 0.01; two-way ANOVA). Interestingly, the SHR OVX group obtained a statistically different greater thickness in trabeculae than the SHR SHAM group with conventional surfaces (*p* = 0.02; Sidak’s test; Figure 2c).

**Figure 3:**
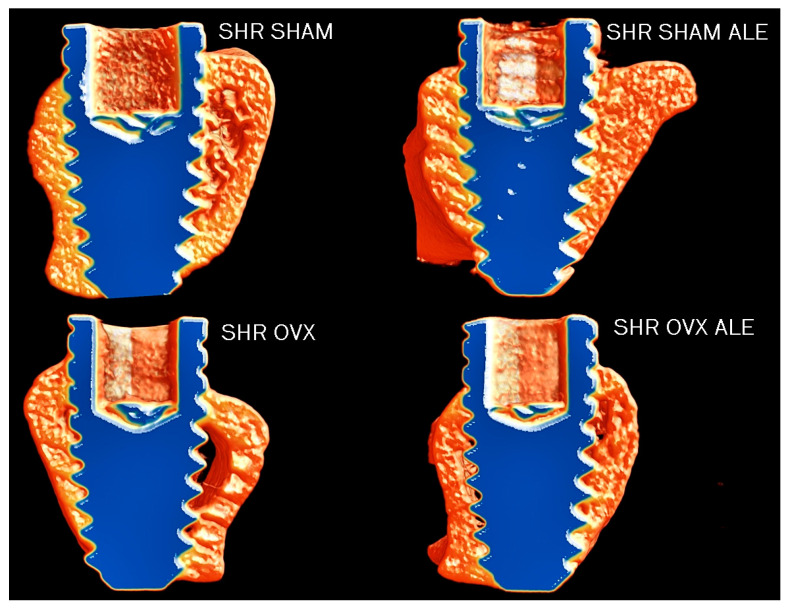
The microtomographic reconstruction of the bicortical implant in the tibia of each group. The microtomography images are representative from all four groups: SHR SHAM, SHR SHAM ALE, SHR OVX, and SHR OVX ALE, respectively. The microtomography images evidenced that estrogen deficiency reduces the peri-implant bone volume in hypertensive ovariectomized rats from the SHR OVX group. The microtomography was executed applying the CTvox software (SkyScan, Version 2.7).

Nevertheless, the number of bone trabeculae decreased in both OVX groups compared to both SHAM groups (*p* < 0.0001; two-way ANOVA; Figure 2d). The catabolic changes by estrogen deficiency caused a higher trabecular separation in both OVX groups (*p* < 0.05; two-way ANOVA; Figure 2e) and elevated the porosity (*p* = 0.0006; two-way ANOVA; Figure 2f) when compared only with the hypertensive animals. The number of trabeculae and the total porosity were statistically different between the groups with implant surfaces with no alendronate (*p* < 0.05; Sidak’s test) and with an alendronate surface (*p* < 0.05; Sidak’s test). Figure 3 contains the microtomography volumetric images of the following groups of samples: SHR SHAM, SHR SHAM ALE, SHR OVX, and SHR OVX ALE. The microtomography structural analysis indicates that estrogen deficiency added to hypertension exerts catabolic effects in the peri-implant bone of hypertensive rats, even with systemic losartan and local alendronate coated on the implant surface.

### 3.4. Epifluorescence Results

**Figure 4:**
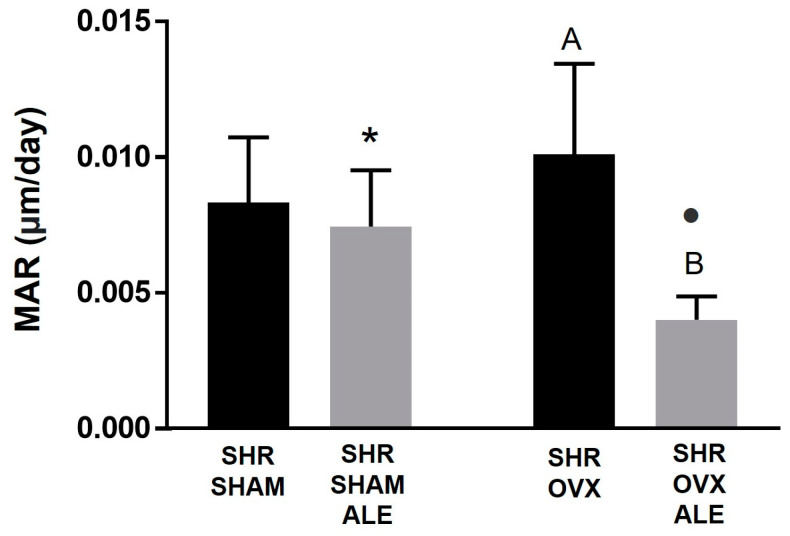
Parameters of epifluorescence evaluated in the peri-implant bone. It was quantified by the daily mineral apposition rate. The different uppercase letters indicate a significant statistical difference between the SHR OVX and SHR OVX ALE groups (*p* < 0.05). In addition, the different symbols “*” and dot demonstrate statistical differences when comparing the SHR SHAM ALE to SHR OVX ALE groups (*p* < 0.05).

In the epifluorescence analysis, it is possible to measure the amount of calcium and alizarin precipitation at the time of the fluorochrome injection (Figure 4). The graph further down shows precipitation at the time of the injection of calcein and alizarin fluorochrome. The daily mineral apposition showed a balance between all evaluated groups. The synergistic action of systemic losartan and local alendronate contributed to the fact that daily calcium precipitation on the peri-implant bone occurred in all groups. It is worth highlighting a slight statistically different reduction in this rate in rats with the implant’s alendronate surface for the SHR SHAM ALE and SHR OVX ALE groups compared to the conventional implant of the SHR SHAM and SHR OVX groups (*p* < 0.0001; two-way ANOVA). Curiously, the trabecular thickness data supported the mineral apposition rate, where the higher MAR value was obtained in the SHR OVX group rather than in the SHR SHAM group, since the SHR OVX group had thicker bone trabeculae than the other groups in a probably compensatory mechanism. The lowest MAR achieved in the SHR OVX ALE group corroborates the smallest trabeculae thickness of them.

**Figure 5:**
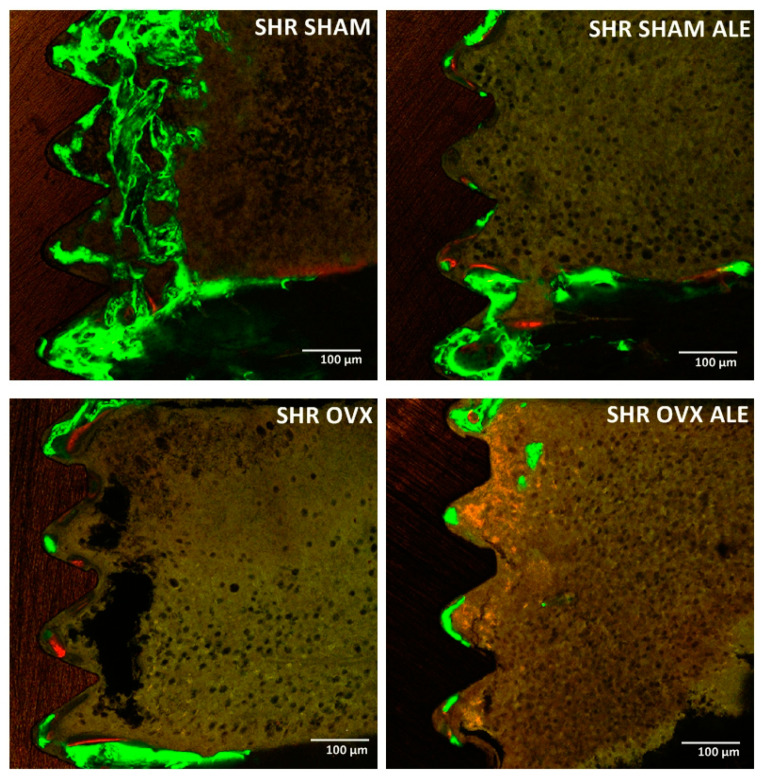
Peri-implant bone dynamics by epifluorescence analysis. Representative figure of the biomarking of the fluorochromes in the peri-implant bone around the implant assessed by the “Color Selection” instrument in the Image J software version 1.53 (Processing Software and Image Analysis, Ontario, ON, Canada). The image represents the overlying of both fluorochromes characterizing the peri-implant bone dynamics by green biomarkers of calcein fluorochrome on the old bone and red biomarkers of the alizarin fluorochrome on the fresh new bone in the SHR SHAM, SHR SHAM ALE, SHR OVX, and SHR OVX ALE groups, respectively.

The descriptive evaluation of Figure 5 shows the peri-implant bone dynamics through the precipitation of fluorochromes in all groups. The fluorochromes are tied to the calcium during their precipitation in the organic bone matrix. Thus, there is an overlapping of green calcein, indicating the previous bone formation, and red alizarin, indicating the new bone formation. The first fluorochrome applied was calcein; therefore, it was the green biomarker of the old bone. Calcein biomarkers were present in all groups, with higher intensity in both SHAM groups, mainly in SHR SHAM, and the lowest in the SHR OVX ALE group. The following administrated fluorochrome was alizarin, so the biomarker in red denoted the fresh newly formed bone. It was also slightly present in all groups.

## 4. Discussion

Untreated hypertension delays alveolar bone healing [36], though antihypertensive drugs can improve bone healing and metabolism [34,37]. Besides hypertension, osteoporosis by estrogen deficiency also impairs alveolar bone healing [33]. Antiresorptive drugs administrated locally, such as sodium alendronate, can improve bone healing during osseointegration [23]. Considering the benefits of antihypertensive and antiresorptive drugs, the hypothesis of the mutually beneficial use of both drugs on postmenopausal bones under hypertensive conditions arise. Interestingly, a prior study showed a higher rate of alveolar bone apposition in hypertensive animals treated with the antihypertensive losartan [31]. Furthermore, untreated hypertensive animals revealed impaired osseointegration [34]. Taking these early results, and considering the replication of a real clinical setting, all hypertensive animals in this study were treated with losartan, since uncontrolled hypertension contraindicates the surgery of implant placement. Together with these propositions, the outcomes revealed a greater reverse torque and higher quality of bone microarchitecture in hypertensive animals than hypertensive and ovariectomized rats, even when treated with alendronate coated on the implant surface and with systemic losartan.

Hypertensive and estrogen-deficient rats confirmed the compromised peri-implant bone microarchitecture established by both conditions. The local administration of alendronate was ineffective in entirely reversing the unfavorable outcome. Also, the antihypertensive treatment did not demonstrate overcoming the consequences of estrogen deficiency on bone, despite its previously established osteoprotective properties in an osteoporotic model [17]. Possibly, the high osteoclastic activity from hypertension [10] and estrogen deficiency rationalizes the nonreversion [38]. The synergism of the drugs worked out in the exclusive presence of hypertension and the absence of estrogen deficiency, where the highest torque was registered. Hypertension leads to an increase in RANKL expression and reduces RUNX2, with a consequent imbalance in the bone metabolism [36]. Osteoporosis also acts by increasing the RANKL and the number of osteoclasts [39]. Therefore, both conditions can decrease calcium deposition in the mineral apposition rate, as shown by epifluorescence. Although alendronate has an affinity to hydroxyapatite crystal and tends to inhibit osteoclast activity [33], the use of ALE was not able to enhance calcium precipitation in hypertensive and ovariectomized animals. By these means, the mineral-apposition-rate values were lower in the SHR OVX ALE group than without ALE. Supporting our results, the mineral apposition rate was lower in animals treated with systemic alendronate through an increase via RANKL and osteoprotegerin [33].

The lower mineral apposition rate observed by fluorochromes in hypertensive and ovariectomized animals can be explained by changes in the calcium metabolism. Increased inflammatory mediators, such as interleukin-6 and tumor necrosis factor, are correlated with the onset of cardiovascular disease in hypertension and osteoporosis [40]. Arteriosclerosis is common in these disorders, causing an intravascular mineralization by a shift of vascular cells into osteoblasts and osteoblast-like cells [1]. Inherently, another study has shown a negative interference in osteogenesis directly by the blood flow [41], which clarifies the lesser percentage of bone volume present in both SHR OVX groups. Insufficient blood supply due to hypertension [42] or estrogen deficiency [41] can be inferred in the presented data. A beneficial effect was revealed on the bone endothelium of elderly mice treated with alendronate [41]. In this previous research, alendronate improved the blood flow and increased endothelial cells, type H osteoprogenitor, and osterix as zinc-finger transcription factor [41]. Feasibly, the alterations in bone vascularization could not be totally reversed with the losartan via the renin–angiotensin system or with the local alendronate in hypertensive and ovariectomized rats.

Postmenopausal women with estrogen deficiency show a rise the expression of type 1 angiotensin II receptors and a decrease estradiol levels, consequently increasing vasoconstriction and blood pressure [5]. Additionally, angiotensin II is considerably associated with an increase in bone resorption [12,18]. However, losartan, as a blocker of angiotensin II, was revealed to improve the mass and strength of bones in osteoporotic rats [43]. Furthermore, this drug increased peri-implant bone thickness in hypertensive rats [34]. The same anabolic effect was found in the alveolar bone mineralization of normotensive rats [31]. In addition, this antihypertensive reduced bone loss in a model of experimental periodontitis [44]. Thus, besides the benefits of losartan in controlling blood pressure and reducing bone loss, it can avoid bone resorption. Although the Tb.Th and calcium precipitation were not compromised in the hypertensive ovariectomized rats, the BV/TV of both SHR OVX groups were lower. The data imply a reduced BV/TV due to decreases in Tb.N and increases in Tb.Sp; the increased Tb.Th can indicate a compensatory mechanism. These results revealed an increased bone loss related to estrogen deficiency. However, the beneficial influence of the local application of alendronate should be stated, since the SHR SHAM ALE and SHR OVX ALE groups showed a slightly higher, but not statistically significant, torque value and bone volume. Thus, alendronate coted on the implant surface may improve, but not totally reverse, this impaired condition by estrogen deficiency.

Previous studies reported inadequate osseointegration in spontaneously hypertensive animals, where losartan proved to be efficient in overcoming this condition [34]. In the present study, losartan used in all groups demonstrated to be ineffective in revering the impaired osseointegration caused by estrogen deficiency in the ovariectomized groups. Hence, the effect of losartan on bone and local alendronate did not effectively reverse the impaired osseointegration in the presence of estrogen deficiency caused by ovariectomy in the hypertension model. Likewise, the use of losartan in synergy with alendronate on the implant surface seems to be a therapeutic strategy for avoiding side effects, such as osteonecrosis. Additionally, new investigations need to consider the local application of alendronate on implants as a treatment strategy for osseointegration in hypertensive females. In this way, the outcomes can also be partially extrapolated to the clinical relevance, where further studies need to elucidate the risk of implants in patients with controlled hypertension and estrogen deficiency. The findings suggest the lack of combined effect between systemic losartan and local alendronate in these simultaneous conditions. In further studies, the concentrations can be adjusted to evaluate new cellular responses in this experimental model. Innovative studies to shift compromised bone healing caused by hypertension and osteoporosis are crucial. Moreover, it is still necessary to verify whether the effect of losartan and alendronate confirmed in hypertensive animals occurs through the vascularization or induction of bone cells.

## 5. Conclusions

In conclusion, the impairment of osseointegration caused by estrogen deficiency in hypertensive rats is characterized by altering biomechanical resistance and peri-implant bone microarchitecture and mineralization. Furthermore, the application of alendronate on the surface of the implants does not fully reverse this impaired condition affected by estrogen deficiency in hypertensive rats.
